# Interaction Between Chronic Endometritis Caused Endometrial Microbiota Disorder and Endometrial Immune Environment Change in Recurrent Implantation Failure

**DOI:** 10.3389/fimmu.2021.748447

**Published:** 2021-10-04

**Authors:** Peigen Chen, Panyu Chen, Yingchun Guo, Cong Fang, Tingting Li

**Affiliations:** Reproductive Medicine Center, The Sixth Affiliated Hospital, Sun Yat-sen University, Guangzhou, China

**Keywords:** chronic endometritis, endometrial microbiota, immune cells, recurrent implantation failure, host-microbiome association

## Abstract

**Objective:**

To investigate the Interaction between chronic endometritis (CE) caused endometrial microbiota disorder and endometrial immune environment change in recurrent implantation failure (RIF).

**Method:**

Transcriptome sequencing analysis of the endometrial of 112 patients was preform by using High-Throughput Sequencing. The endometrial microbiota of 43 patients was analyzed by using 16s rRNA sequencing technology.

**Result:**

In host endometrium, CD4 T cell and macrophage exhibited significant differences abundance between CE and non-CE patients. The enrichment analysis indicated differentially expressed genes mainly enriched in immune-related functional terms. *Phyllobacterium* and *Sphingomonas* were significantly high infiltration in CE patients, and active in pathways related to carbohydrate metabolism and/or fat metabolism. The increased synthesis of lipopolysaccharide, an important immunomodulator, was the result of microbial disorders in the endometrium.

**Conclusion:**

The composition of endometrial microorganisms in CE and non-CE patients were significantly different. *Phyllobacterium* and *Sphingomonas* mainly regulated immune cells by interfering with the process of carbohydrate metabolism and/or fat metabolism in the endometrium. CE endometrial microorganisms might regulate Th17 response and the ratio of Th1 to Th17 through lipopolysaccharide (LPS).

## Introduction

Recurrent implantation failure (RIF) is usually defined as a woman under 40 who has received at least 4 high-quality embryos in at least three fresh or frozen cycles or has transferred more than 10 high-quality embryos and still cannot obtain a clinical pregnancy (1–3). Recent studies had confirmed that chronic endometritis (CE) was an important cause of recurrent reproductive failure (included recurrent miscarriage and recurrent implantation failure) (4). Chronic endometritis (CE) was a persistent inflammation of the endometrium, and CD138 immunohistochemical (IHC) staining of plasma cells was a more accurate and sensitive diagnostic method for the diagnosis of CE (5–9).

Multiple studies had shown that a good balance of innate and adaptive local and peripheral immune systems was the basis for a successful pregnancy (10, 11). The study by Yuye Li et al. showed that the abundance of endometrial immune cells in CE patients was increased, and the high abundance of immune cells might be related to decreased endometrial receptivity and repeated pregnancy failures (4).

It was now clear that microorganisms affect immunity (12, 13). It also knew that disturbances in the balance between the microbiota and the immune system might lead to inappropriate immune responses or excessive inflammation, or down-regulation of the immune response and pathogenic bacteria’s dominance of normal symbionts “ecological disorders” (14). However, the mechanism of action of endometrial microbiota in RIF patients with CE remains unclear. The purpose of this study was to explore the regulation of endometrial microbes on the endometrial immune cells of RIF patients with CE.

## Method

### Collection of Research Subjects and Ethical Approval

The RIF cohort selected patients who underwent frozen embryo transfer (FET) in Reproductive Medicine Center, The Sixth Affiliated Hospital, Sun Yat-sen University, with the inclusion criteria. (a). At least four good-quality embryos were accepted in at least three fresh or frozen cycles, but still not pregnant. (b). Normal ovarian reserve function (levels of follicle stimulation hormone (FSH) <12 mIU/ml and AMH >1.1 ng/ml). (c). Confirmed by the examination (ultrasound or hysteroscopy) confirms that the uterine cavity was in normal shape. (d). Normal chromosome karyotype. (e). No antibiotics were received in the month before collection. (f). All patients were examined for cervical and vaginal secretions one month and one week before the embryo transplant to rule out infectious diseases.

The Ethics Committee approved the study of Sixth Affiliated Hospital of Sun Yat-Sen University. All patients were fully informed and voluntarily participate in the study and sign an informed consent form. At the same time, this study did not cover any research products.

### Sample Collection Procession

Endometrial specimens were collected on LH+7 (natural cycles) or P+5 (artificial hormone cycle) according to the process (described in **Supplementary Data Sheet 1**) and stored at -20°C.

### Host RNA Extraction and Sequencing

After completing the library construction according to the manufacturer’s instructions (as described in **Supplementary Data Sheet 1**), pair-end sequencing was performed on the HiSeq 2500 platform (Illumina).

### Host RNA-Seq Analysis

FASTQ files were processed by fastp (15), Bowite2 (16), HISAT2 (17) and StringTie (18, 19) to get the count matrix (as described in **Supplementary Data Sheet 1**). The human genome GRCh38 from Gencode v26 was used as the reference genome. Then the count value of gene expression was normalized to TPM (Transcripts Per Kilobase of exon model per Million mapped reads) value.

### Selection of Differentially Expressed Genes and Functional Enrichment Analysis

The differentially expressed genes between CE and non-CE patients were selected by using DESeq2 (20) R package with the p value < 0.05 and log fold change >1 as cut-off. Then functional enrichment analysis of DEGs was performed by clusterprofiler R package (21) and OmicShare tool (http://www.omicshare.com/tools).

### Calculation of Endometrial Immune Cells Infiltration

CIBERSORT is a tool that can estimate the abundances of 22 types of immune cells, including seven T cell types, naïve and memory B cells, plasma cells, NK cells, and myeloid subsets (22). By using CIBERSORT, the infiltration of immune cells in endometrium was calculated. To understand the consistence of T cells, we also used ImmuCellAI web tools which included 18 T-cell subtypes (23) to calculate the infiltration of subtypes of T-cells.

### 16S rRNA Extraction and Sequencing

After completing the library construction according to the manufacturer’s instructions with the universal primers 341F (5′-CCTACGGGNGGCWGCAG-3′)and 806R(5′-GGACTACHVGGGTWTCTAAT-3′)(as described in **Supplementary Data Sheet 1**), NovaSeq 6000 was used for high throughput sequencing.

### Endometrial Microbiota Analysis

Endometrial microbiota analysis was processed following QIIME2 analysis pipeline (as described in **Supplementary Data Sheet 1**) (24).

The differential abundance of endometrial microbiota was selected using LEfSe (Linear discriminant analysis Effect Size) (25).

PICRUSt2 (Phylogenetic Investigation of Communities by Reconstruction of Unobserved States) (26) was used to predict the functional profiles of the endometrial microbes. Different metacyc (27) functional profiles were screened by STAMP software (version 2.1.3) (28).

## Results

### Endometrial Transcriptome Analysis

**Figure 1:**
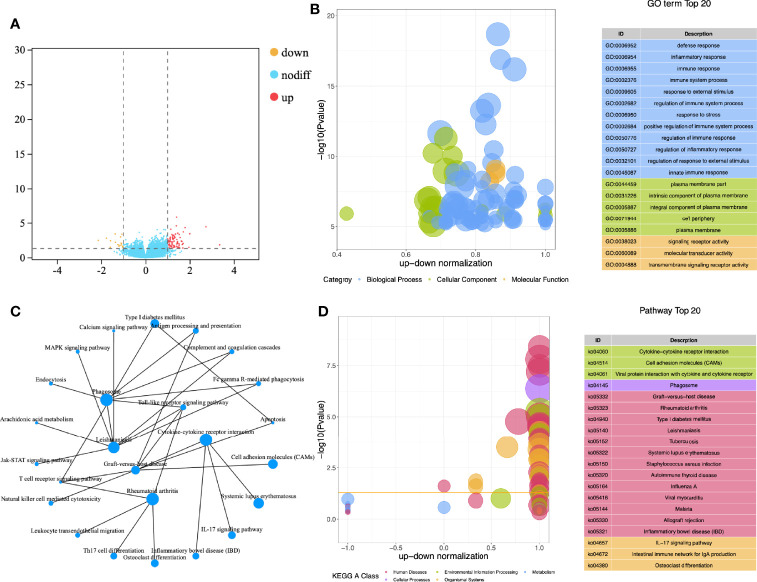
Functional enrichment analysis of DEGs. **(A)** Volcano map for differential expression analysis; **(B)** Z-score bubble chart of Gene Ontology (GO) enrichment analysis; **(C)** Network diagram of KEGG enrichment analysis; **(D)** Z-score bubble chart of KEGG enrichment analysis.

A total of 112 subjects (included 32 CE patients and 72 non-CE patients) were recruited in this study. After obtaining the gene expression matrix, we obtained 93 differentially expressed genes through DESeq2 (logFC ≥ |1|, *p* < 0,05 [**Figure 1A**, **Supplementary Table 1**)]. By functional enrichment analysis, we found that DEGs were mainly enriched in the immune and inflammatory-related GO (Gene Ontology) terms, such as immune response (GO:0006955), immune system process (GO:0002376), and inflammatory response (GO: 0006954) (**Figure 1B**, **Supplementary Table 2**). The top 20 enriched pathways were showed in **Figure 1D** and the interaction network of these pathways was described in **Figure 1C**. Most of these pathways were also related to inflammation and immune processes in the endometrium.

### Calculation of Endometrial Immune Cells Infiltration

**Figure 2:**
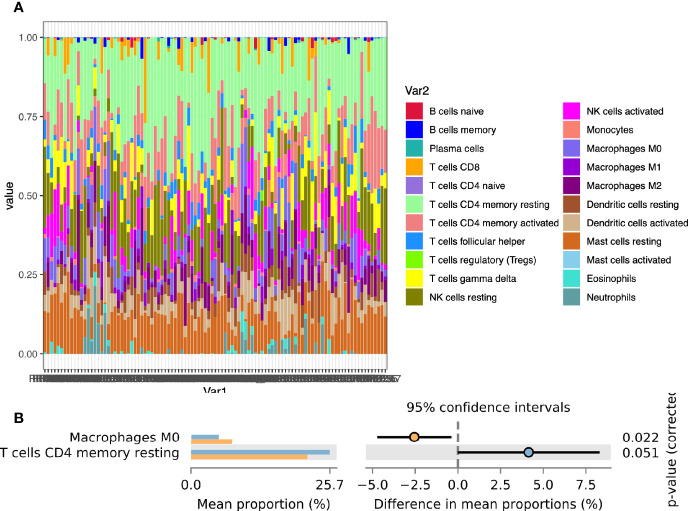
Calculation of endometrial immune cells infiltration. **(A)** The composition of 22 immune cells in the CE group and the non-CE group; **(B)** Comparison of differences in immune cell immersion.

To further explore the infiltration of immune cells in the endometrium, we used COBERSORT to calculate the degree of infiltration of 22 immune cells (**Figure 2A**). The infiltration of memory CD4 T cells and Macrophages was a significant difference between the two groups (**Figure 2B**). We also used ImmuCellAI to estimate the infiltration of 18 subtypes of T cells (**Supplementary Table 3**).

### Endometrial Microbiota Analysis

**Figure 3:**
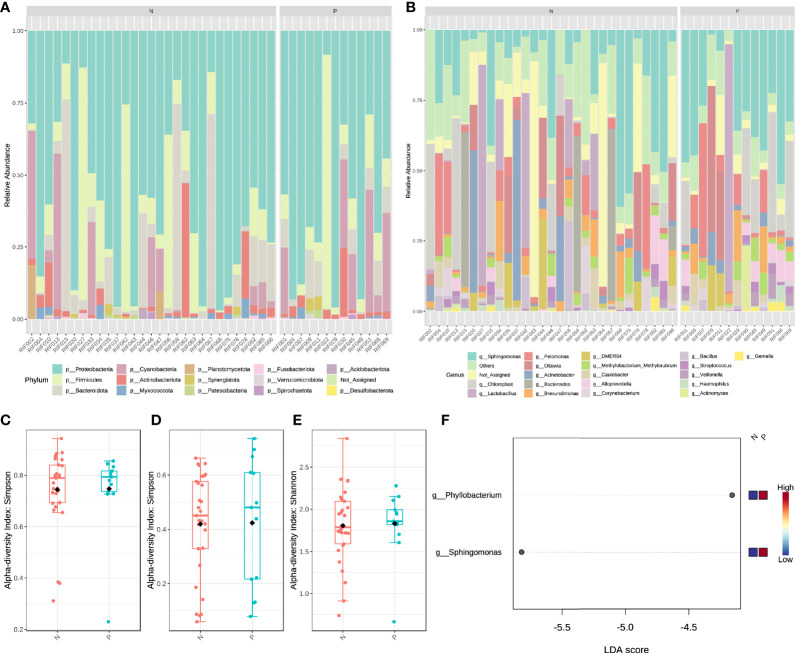
The structural characteristics of the endometrial microbiota. Taxonomic classification of the endometrial microbiota of CE (P) and non-CE (N) group at the level of Phylum **(A)** and Genus **(B)**. The α diversity calculated by Simpson index of the endometrium microbiota between CE and non-CE group at the level of feature **(C)**, Phylum **(D)** and Genus **(E)**. **(F)** Linear discriminant analysis of the differential abundance endometrial microbiota between CE and non-CE group.

We performed 16s rRNA sequencing on the endometrial microbiota of 43 patients (14 CE patients and 29 non-CE patients). As the rarefaction curve was shown (**Supplementary Figure 1**), the 16s rRNA sequencing in this study had sufficient sequencing depth. The top 15 phylum and top 20 Genus were shown in **Figures 3A, B**. The α diversity of the endometrium microbiota calculated by Simpson showed that there was no significant difference in species diversity between the two groups of feature level (**Figure 3C**), phylum level (**Figure 3D**), and genus level (**Figure 3E**) (T-test).

Based on the results of LEfSe (LDA ≥ 2), the abundance of *Phyllobacterium* and *Sphingomonas* was significantly increased in CE patients (**Figure 3F**). Spearman correlation analysis was performed by using the cor. test() function with a two-sided alternative hypothesis to analyze the relationship between the immune cells and genus selected by LEfSe. Compared with other methods (such as Pearson), the normalized count (gene expression) and component data (relative abundance of microbiota) of Spearman’s correlation analysis performed better (29). Cytoscape software (30) was used to visualize the results of the analysis.

**Figure 4:**
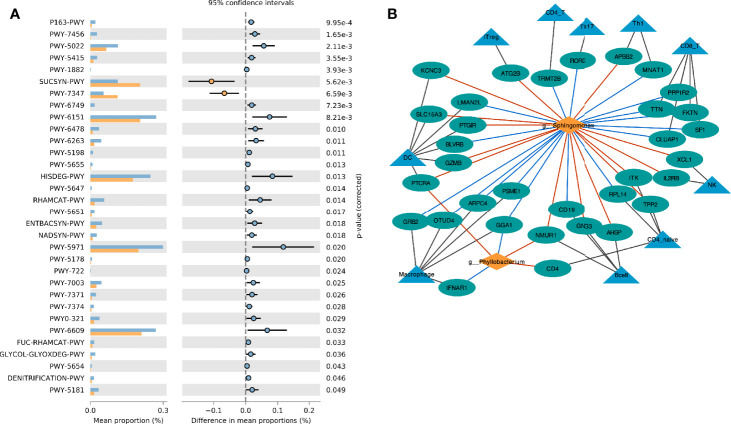
Interaction between endometrial immune cell and microbiota. **(A)** Comparison of differences in metabolic pathway between CE and non-CE group; **(B)** Network visualizing the relationship between genus, immune cell marker genes and immune cells. The red line indicates the positive correlation (red) or negative (blue) relationship between the genera and the gene.

Based on the Metacyc database, PICRUST2 was used to analyze the metabolic function of the endometrial microbiota. Through the STAMP software, we found that the CE group was mainly enriched in the sucrose biosynthesis III pathway (PWY-7347) and sucrose biosynthesis I pathway (SUCSYN-PWY) (Welch’s t-test, *p* < 0.05, **Figure 4A** and **Supplementary Table 4**).

### Interaction Between Endometrial Immune Cell and Microbiota

The marker genes of immune cells were obtained from a previous study (23). The correlation network between the selected Genus and the marker genes of immune cells was constructed by Spearman analysis (**Figure 4B**). We found that *Phyllobacterium* and *Sphingomonas* had similar relationships with immune cells, and they were mainly positively related to dendritic cells (DC), natural killer (NK) cells, iTreg cells and B cells. These two genera were negatively correlated to macrophage cells.

**Figure 5:**
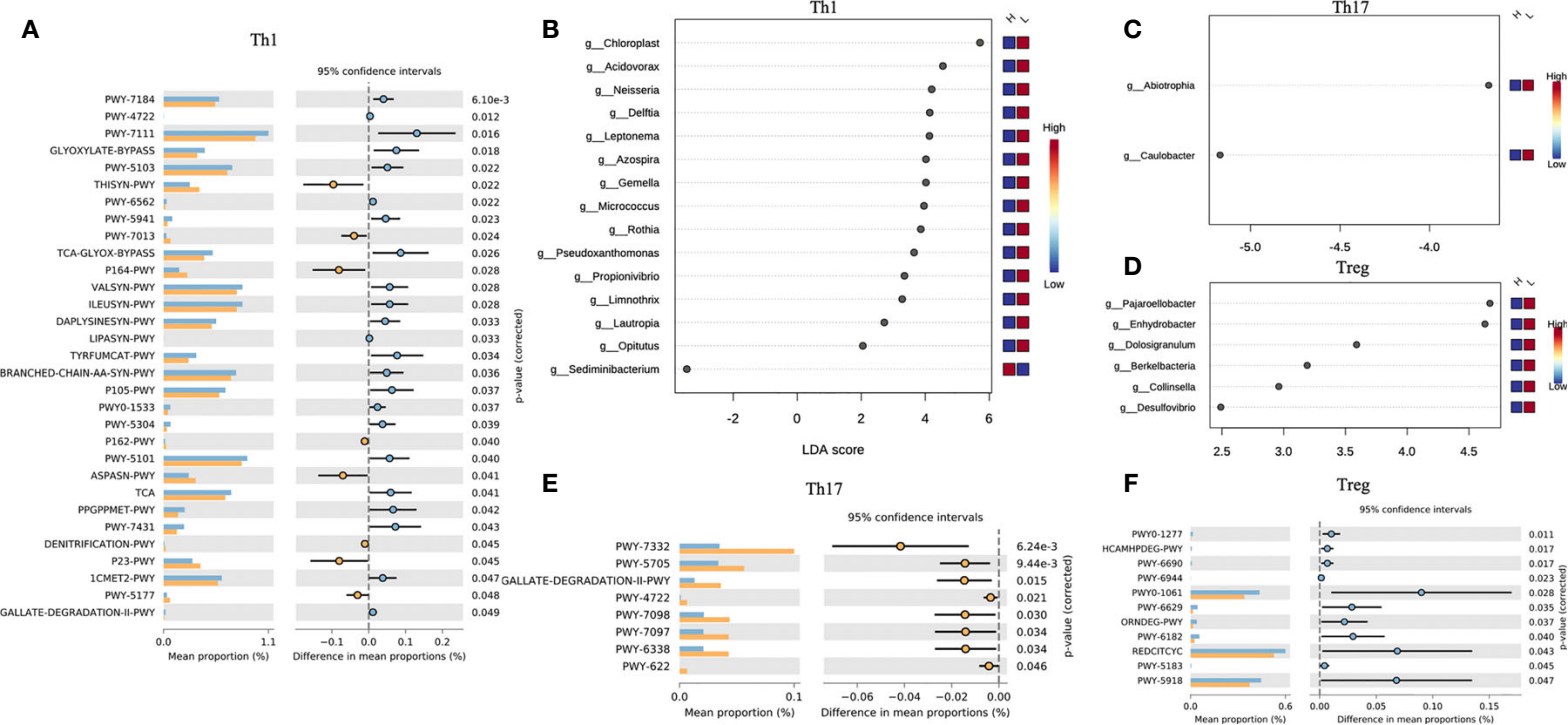
Interaction between T cell subtypes and microbiota. Comparison of differences in metabolic pathway of Th1 **(A)**, Th17 **(E)**, and Treg **(F)**. Different genera related to Th1 **(B)**, Th17 **(C)**, Treg **(D)** abundance.

In the host’s pathway analysis, T cell-related signaling pathways show an important position. After calculating the abundance of 18 T cell subtypes using ImmuCellAI, we analyzed the related genus of several subtypes reported in other studies (14) included Th1, Th17, and T regular (Treg) (**Figure 5** and **Supplementary Table 5**). Endometrial microbiota in patients with high Th1 abundance (**Figure 5B**) was active in several glycolysis-related pathways, including super pathway of thiamine diphosphate biosynthesis I (THISYN-PWY), reductive TCA cycle I (P23-PWY), super pathway of L-aspartate and L-asparagine biosynthesis (ASPASN-PWY) and purine nucleobases degradation I (P164-PWY) (**Figure 5A**). The PWY-7332 pathway, which was highly active in the endometrium of patients with high abundance of Th17, was a super pathway for the synthesis of Lipopolysaccharides (LPS), which is an important immunomodulator (**Figures 5C, E**).

## Discussion

Chronic endometritis (CE) is an important factor for recurrent implantation failure (31). The endometrium of CE patients showed a high abundance of immune cell infiltration, which would cause a decrease in the receptivity of the endometrium, an important feature of recurrent implantation failure (4). It has been proven that immune adaptation, one of the key mechanisms for the establishment and maintenance of pregnancy, was directly affected by local microorganisms (32). In this study, we combined the endometrial transcriptome and microbial diversity analysis to illustrate that endometrial microbiota regulated the abundance of endometrial immune cells through metabolic activities.

The functional enrichment analysis of differential genes in the endometrial transcriptome showed that the functions of DEGs were mainly enriched in immune and inflammation-related items. In the network diagram of enriched pathways, cytokine-cytokine receptor interaction and Toll-like receptor signaling pathway were the central pathways in the network. Toll-like receptors (TLRs) are the first line of defense against pathogen invasion and play a key role in inflammation, immune cell regulation, survival, and proliferation (33). This is the result of the body’s defense during endometritis. At the same time, this pathway and cytokine-cytokine receptor interaction played an important role in the regulation of T cell plasticity and differentiation (33, 34). After calculating the infiltration of endometrial immune cells by CIBERSORT, we found that resting CD4 memory T cells was high infiltration in the non-CE group. This might indicate that in CE, CD4 T cells are significantly activated due to inflammation. This was consistent with the conclusion of another study (4).

It has been proven that bacteria can affect immunity (12, 13). We introduced 16s rRNA sequencing technology to analyze the impact of endometrial microbiota on immune cells. The results of LEfSe showed that the abundance of *Phyllobacterium* and *Sphingomonas* in the CE group was significantly higher than that in the non-CE group. In the network diagram of the relationship between different genus and immune cell marker genes, we found that *Phyllobacterium* and *Sphingomonas* had a significant positive correlation with B cells. Since the infiltration of plasma cells in the endometrial stroma was considered a characteristic of endometritis (35), it confirmed that these two different genera played an important role in endometritis.

At the same time, we noticed a significant positive correlation between *Sphingomonas* and uterus NK (uNK) cells. In addition, in the differential analysis of the transcriptome, the expression of CD16 (FcγRIII) in the CE group was significantly higher than that in the non-CE group. CD16+ is a characteristic of the uNK cell population that specifically kills infected cells (36). The abnormal differentiation of uNK cells might lead to embryo implantation failure through the development of endometrial and/or decidual blood vessels and trophoblasts (37). The study of May-Tal Sauerbrun-Cutler et al. showed that a higher level of CD16 + uNK cells was a risk factor for embryo transfer failure in infertile women before frozen embryo transfer (38). Macrophage was known as a common anti-epidemic presenting cell (APC). It was the main cytokine producer in the human endometrium (39) and played an important role in the establishment of endometrial receptivity (40). The results of this study suggested that *Phyllobacterium* and *Sphingomonas* were significantly negatively correlated with macrophages. This might be one of the important mechanisms of endometrial microbiota affecting endometrial receptivity.

It was known that metabolic activity had an important regulatory effect on immune cells (41, 42). Endometrial microbiota was metabolically active, and the crosstalk of metabolic activity might be an important way for endometrial microbiota to interact with endometrial immune cells. In the study, two metabolic pathways were highly active in the CE group, PWY-7347, and SUCSYN-PWY. SUCSYN-PWY described the process of generating sucrose by *phosphoglycerol* and *3-phospho-D-glyceric acid*. The intermediate products included *UDP-α-D-glucose*. PWY-7347 described the process of *glucose-6-phosphate* and *UDP-α-D-glucose* to produce sucrose, and phosphate and UDP were produced in the middle. Obviously, these two metabolic pathways were interaction with glucose and lipid metabolism, which had been proved to be closely related to the regulation of T cells and macrophages (41, 42). These active microbiotas competed for energy materials of the host (including endometrial cells and immune cells) by consuming intermediate products of glycolysis.

Th1 is a subtype of CD4 T cells, and its increased abundance was considered to be unfavorable for embryo implantation (43). In this study, we found that endometrial microorganisms with high Th1 abundance exhibited significant-high activity in many important metabolic pathways, including L-aspartic acid and L-asparagine biosynthesis, reducing TCA cycle, degradation of purine nucleobases, and biosynthesis of thiamine diphosphate. Obviously, this was related to multiple links in carbohydrate metabolism (including glycolysis). Therefore, the abnormal activities of the microbiota in the endometrium might regulate the conversion of Th1/Th2 by interfering with the process of carbohydrate metabolism and/or fat metabolism. In addition, Th17 and Treg also played an important role in the regulation of immune adaptation during embryo implantation, and the imbalance of Th17/Treg ratio would have an adverse effect on embryo implantation. In the study of intestinal microbes, the researchers confirmed that lipopolysaccharide (LPS) could induce a decrease in the number of Tregs and an increase in the number of Th17 and Th1 (44). In our study, the endometrial microbiota of patients with high Th17 abundance was significantly active in the relevant pathways of LPS synthesis. It meant that the imbalance in the number and proportion of immune cells in the endometrium may be caused by a similar mechanism.

## Conclusion

In this study, we combined the transcriptome and 16s rRNA sequencing technology to analyze the interaction between the endometrium microbial disorder caused by chronic endometritis and the immune cells in the endometrium of patients with recurrent implantation failure. We found that the composition of endometrial microorganisms in CE and non-CE patients were significantly different. *Phyllobacterium* and *Sphingomonas* mainly regulated immune cells by interfering with the process of carbohydrate metabolism and/or fat metabolism in the endometrium. The results of this study supplemented part of the mechanism of microbial regulation of the immune environment of the endometrium.

## Data Availability Statement

The transcriptome sequencing for 112 endometrium samples have been deposited with the National Center for Biotechnology Information (NCBI) under reference number PRJNA747622. The 16s rRNA gene sequencing for 43 endometrial microbiota samples have been deposited with the NCBI under reference number PRJNA732058.

## Ethics Statement

The studies involving human participants were reviewed and approved by Sixth Affiliated Hospital of Sun Yat-Sen University. The patients/participants provided their written informed consent to participate in this study.

## Author Contributions

CF, TL, and PeC carried out the study. PeC and TL analyzed and interpreted the data and drafted the manuscript. YG and PaC collected and followed up the samples. TL and CF revised the manuscript. CF, TL, and PeC coordinated the study, participated in the design, and reviewed the manuscript. All authors contributed to the article and approved the submitted version.

## Funding

This work was supported by the National Natural Science Foundation of China (grant no. 81871214), the National Key R&D Program of China (grant no. 2017YFC1001603), and the National Natural Science Foundation of China (grant no. 81070495).

## Conflict of Interest

The authors declare that the research was conducted in the absence of any commercial or financial relationships that could be construed as a potential conflict of interest.

## Publisher’s Note

All claims expressed in this article are solely those of the authors and do not necessarily represent those of their affiliated organizations, or those of the publisher, the editors and the reviewers. Any product that may be evaluated in this article, or claim that may be made by its manufacturer, is not guaranteed or endorsed by the publisher.

## Supplementary Material

Supplementary Figure 1 The rarefaction curve of 16s rRNA data.

Supplement Data Sheet 1 Detailed description of Methods.

Supplementary Table 1 Differentially expressed genes of DESeq2.

Supplementary Table 2 The result table of functional enrichment analysis of DEGs.

Supplementary Table 3 Infiltration of 18 subtypes of T cells.

Supplementary Table 4 Comparison Results Table of PICRUST2 Metabolic Pathway.

Supplementary Table 5 Comparison Results Table of PICRUST2 Metabolic Pathway of Th1, Th17, and Treg.
