# Supplementary figures and images for: Interaction Between Chronic Endometritis Caused Endometrial Microbiota Disorder and Endometrial Immune Environment Change in Recurrent Implantation Failure
Source: Front Immunol. 2021 Oct 4;12:748447. doi: 10.3389/fimmu.2021.748447 (PMC8521098; doi:10.3389/fimmu.2021.748447)

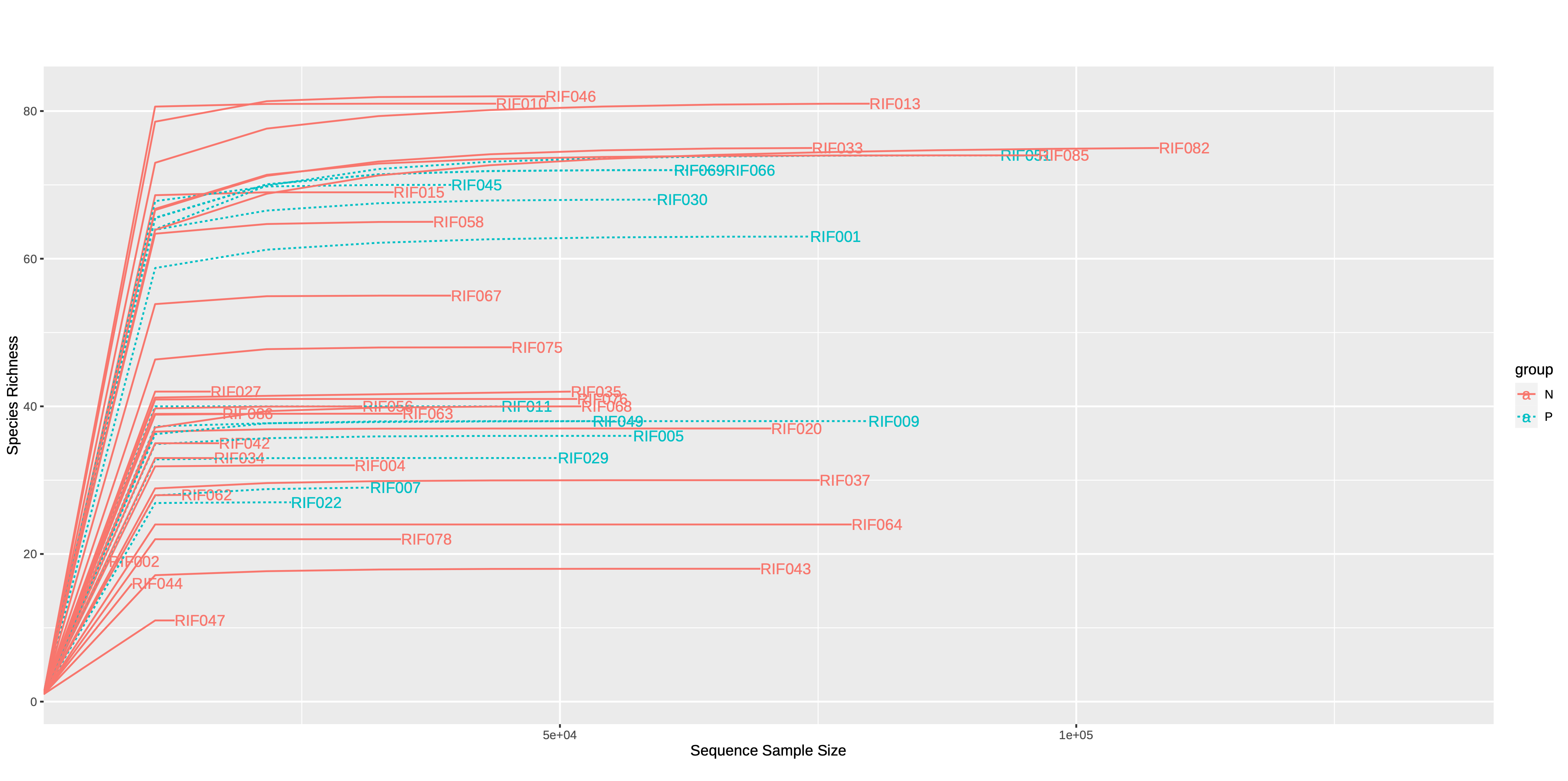

Supplement: Supplementary Figure 1 — The rarefaction curve of 16s rRNA data. [file Image_1.tif]
