# Supplementary material for: Interaction Between Chronic Endometritis Caused Endometrial Microbiota Disorder and Endometrial Immune Environment Change in Recurrent Implantation Failure
Source: Front Immunol. 2021 Oct 4;12:748447. doi: 10.3389/fimmu.2021.748447 (PMC8521098; doi:10.3389/fimmu.2021.748447)
Supplement: Supplement Data Sheet 1 — Detailed description of Methods. [file DataSheet_1.docx]

**Method**

**Sample collection procession**

Endometrial specimens were collected on LH+7 (natural cycles) or P+5 (artificial hormone cycle) by using a sterile endometrium sampler kit with a separate package (Type I, Run Ting). The sampler was divided into inner and outer layers. The inner layer was a soft long-handled swab with a small ball on the front end, and the outer layer was a transparent protective cover to prevent the swab from being contaminated by vagina and cervix. During the operation, we first disinfected the vulva and exposed the cervix with a vaginal speculum, then we disinfected the cervix and cervical canal and gently inserted the sampler along the direction of the uterus under the ultrasound. We observed that the front-end ball reached the internal cervix, kept the outer layer still, and extended the inner swab. When the sampling was completed, the swab was first withdrawn and then withdrawn from the outer layer. The sample was carefully packed in Corning® 2 mL External Threaded Polypropylene Cryogenic Vial (430659, CORNING Inc. USA) and quickly transferred to liquid nitrogen.

The sampling process of endometrium was similar to a disposable sterile endometrial sampler (Yikon Inc.). With this method, endometrial tissue was obtained by suction and placed in the tissue preservation solution (XK-039-3, Yikon Inc.), then placed at -20℃.

**Host RNA extraction and sequencing**

According to the manufacturer's instructions, total RNA was extracted using RNeasy Micro Kit (74004, Qiagen). Then, Quabit HS RNA Kit (Q32855; Thermo Fisher Scientific) was used for RNA quantitative detection. Then Agilent Bioanalyzer 2100 (Agilent) was used to check the integrity of the extracted total RNA. Samples with RNA integrity index (RIN) > 7 were considered qualified samples and used for subsequent testing.

Next, RNA reverse transcription and amplification were performed using MALBAC® Platinum Single Cell RNA Amplification Kit (KT110700796; Xukang Co., Ltd.). The positive and negative controls were 500 ng of high-quality host total RNA and ultrapure water, respectively. After the reverse transcription was completed, 1uL cDNA was taken for 10-fold dilution. Then Agilent Bioanalyzer 2100 (Agilent) was used for detection.

Finally, the library was constructed using gene sequencing and library preparation kit (XY045, Xukang Co., Ltd.). After purification, the library was quantified with Qubit dsDNA HS kit (Q32584, Invitrogen). According to the quantitative results of Qubit, each sample was taken from 10ng library and mixed in equal proportions. The mixed library was subjected to Qubit quantitative detection again. Then, pair-end sequencing was performed on the HiSeq 2500 platform (Illumina).

**Host RNA-seq analysis**

FASTQ files were processed by using fastp [1] to remove reads containing adapters, more than 10 % unknown nucleotides (N), and more than 50% of low quality (Q-value≤20) bases. Bowtie2 [2] was then used to map the short reads to ribosome RNA (rRNA) database to remove them. Next, the paired-end clean reads were mapped to the human genome GRCh38 from Gencode v26 using HISAT2 [3] with “-rna-strandness RF." Finally, StringTie [4, 5] was used to assemble the mapped reads. Then the count value of gene expression was normalized to TPM (Transcripts Per Kilobase of exon model per Million mapped reads) value.

**16S rRNA extraction and sequencing**

DNA was extracted and purified using QIAamp DNA Microbiome Kit (51704, Qiagen) according to the manufacturers' instructions and then quantified by using Nanodrop 2000. Next, we used 20 ng DNA as a template to amplify the V3-V4 region of the 16S rRNA gene using PCR with the universal primers 341F (5′-CCTACGGGNGGCWGCAG-3′）and 806R（5′-GGACTACHVGGGTWTCTAAT-3′） by using 2×HiFi PCR Mix kit (CW2648M, CWbio Inc.). The PCR reaction conditions were as follows: denaturation at 95°C (5 min), 25 cycles of denaturation at 95°C (30 s), annealing at 55°C (30 s), and elongation at 72°C (45 s), and extension at 72°C (5 min). After PCR amplification was completed, amplified products were purified using DNA Clean-up Kit (CW2301, CWbio Inc.). Next, the library was prepared using NEBNext Ultra™ II DNA Library Prep Kit (E7645, NEB). Finally, NovaSeq 6000 was used for high throughput sequencing.

**Endometrial microbiota analysis**

FASTQ files were processed by using FastQC [6] to perform quality control. After quality control, the ambiguous bases (N) of paired end reads and primer were cut off by Trimmomatic [7]. The low-quality sequences with average quality scores below 20 were also cut off by sliding window trimming approach of Trimmimatic. Next, pair-end reads were assembled by FLASH software with 10bp of minimal overlapping, 200bp of maximum overlapping, and 20% maximum mismatch rate [8].

**Endometrial microbiota analysis**

Endometrial microbiota analysis was processed following QIIME2 analysis pipline [22]. After importing the original sequence data, the q2-demux plug-in was used for quality control and filtering, and then DADA2 [23] (via q2-dada2) was used for denoising. Phylogeny was constructed by using fasttree2 (via q2‐phylogeny) [24]. Alpha‐diversity metrics (observed OTUs and Faith’s Phylogenetic Diversity [25]), beta diversity metrics (weighted UniFrac [26], unweighted UniFrac [27], Jaccard distance, and Bray‐Curtis dissimilarity), and Principal Coordinate Analysis (PCoA) were estimated using q2‐diversity. The q2‐feature‐classifier [28] classify‐sklearn naïve Bayes taxonomy classifier was then used to assign taxonomy to amplicon sequence variants (ASVs) according to the silva 138 reference sequences [29].

The differential abundance of endometrial microbiota was selected using LEfSe (Linear discriminant analysis Effect Size) [30].

PICRUSt2 (Phylogenetic Investigation of Communities by Reconstruction of Unobserved States) [31] was used to predict the functional profiles of the endometrial microbes. Different metacyc [32] functional profiles were screened by STAMP software (version 2.1.3) [33].

1. Chen, S., et al., *fastp: an ultra-fast all-in-one FASTQ preprocessor.* Bioinformatics, 2018. **34**(17): p. i884-i890.

2. Langmead, B. and S.L. Salzberg, *Fast gapped-read alignment with Bowtie 2.* Nat Methods, 2012. **9**(4): p. 357-9.

3. Kim, D., B. Langmead, and S.L. Salzberg, *HISAT: a fast spliced aligner with low memory requirements.* Nat Methods, 2015. **12**(4): p. 357-60.

4. Pertea, M., et al., *StringTie enables improved reconstruction of a transcriptome from RNA-seq reads.* Nat Biotechnol, 2015. **33**(3): p. 290-5.

5. Pertea, M., et al., *Transcript-level expression analysis of RNA-seq experiments with HISAT, StringTie and Ballgown.* Nat Protoc, 2016. **11**(9): p. 1650-67.

6. S., A., *FastQC: a quality control tool for high throughput sequence data*. 2010.

7. Bolger, A.M., M. Lohse, and B. Usadel, *Trimmomatic: a flexible trimmer for Illumina sequence data.* Bioinformatics, 2014. **30**(15): p. 2114-20.

8. Reyon, D., et al., *FLASH assembly of TALENs for high-throughput genome editing.* Nat Biotechnol, 2012. **30**(5): p. 460-5.

9. Hill, T.C., et al., *Using ecological diversity measures with bacterial communities.* FEMS Microbiol Ecol, 2003. **43**(1): p. 1-11.

10. Chao, A. and J. Bunge, *Estimating the number of species in a stochastic abundance model.* Biometrics, 2002. **58**(3): p. 531-9.

11. McMurdie, P.J., *phyloseq: An R package for reproducible interactive analysis and graphics of microbiome census data*. 2013.

12. Chong, J., et al., *Using MicrobiomeAnalyst for comprehensive statistical, functional, and meta-analysis of microbiome data.* Nat Protoc, 2020. **15**(3): p. 799-821.

13. Dhariwal, A., et al., *MicrobiomeAnalyst: a web-based tool for comprehensive statistical, visual and meta-analysis of microbiome data.* Nucleic Acids Res, 2017. **45**(W1): p. W180-W188.

14. Segata, N., et al., *Metagenomic biomarker discovery and explanation.* Genome Biol, 2011. **12**(6): p. R60.

15. Douglas, G.M., et al., *PICRUSt2 for prediction of metagenome functions.* Nat Biotechnol, 2020. **38**(6): p. 685-688.

16. Caspi, R., et al., *The MetaCyc database of metabolic pathways and enzymes - a 2019 update.* Nucleic Acids Res, 2020. **48**(D1): p. D445-D453.

17. Parks, D.H., et al., *STAMP: statistical analysis of taxonomic and functional profiles.* Bioinformatics, 2014. **30**(21): p. 3123-4.
